# Association between baseline cognition and clinical response in first-episode schizophrenia

**DOI:** 10.1186/s12888-026-08481-4

**Published:** 2026-08-05

**Authors:** Yuanyuan Zhu, Ye Yang, Renrong Wu, Dong Cao, Fuli Jiang

**Affiliations:** 1 Department of Psychiatry, The Affiliated Brain Hospital of Nanjing Medical University, Nanjing Brain Hospital, Nanjing, Jiangsu 210029 PR China; 2 Department of Psychiatry, National Clinical Research Center for Mental Disorders, and National Center for Mental DisordersThe Second Xiangya Hospital of Central South University, Changsha, Hunan 410011 China

**Keywords:** Cognition, Association, Clinical response, Schizophrenia

## Abstract

**Background:**

This study aimed to investigate the association between baseline cognitive performance and the clinical response to antipsychotics in first-episode schizophrenia (FES) patients.

**Methods:**

This study examined 769 patients from a multi-center research cohort. Neurocognition was measured using the MATRICS Consensus Cognitive Battery (MCCB) in schizophrenia at baseline. Clinical response was defined as ≥ 50% reduction in PANSS total score from baseline to week 8. Pearson correlation analysis was performed for preliminary exploratory assessment. Multivariable logistic regression models (response: yes/no) were fitted with each cognitive domain as dependent variable, adjusted confounding factors. To test effect modification, we added interaction terms: (i) cognition × antipsychotic type and (ii) cognition × baseline illness severity. For domains with significant interactions (*P* < 0.05), stratified analyses were performed within each drug group and severity stratum.

**Results:**

Logistic regression analysis demonstrated that all baseline neurocognitive domains were significantly associated with clinical response (All *P* < 0.05). Significant interactions between neurocognitive performance and antipsychotics were observed across all domains except Working Memory and Reasoning/problem solving. Similarly, significant interactions between neurocognition and baseline illness severity were found for all domains except Visual and Verbal learning. Further stratified analyses revealed that baseline cognitive performance was associated to clinical response to amisulpride and risperidone. Additionally, significant associations between neurocognition and clinical response were consistently observed in patients with moderate-to-severe illness severity across all domains, with the exception of Visual and Verbal Learning.

**Conclusions:**

The association between baseline cognitive performance and treatment response is contingent on medication regimen and baseline illness severity.

**Trial registration:**

The study was registered on ClinicalTrials.gov (NCT03451734) with a registration date of January 23, 2018.

**Supplementary Information:**

The online version contains supplementary material available at https://doi.org/10.1186/s12888-026-08481-4.

## Background

Cognitive deficit has been considered a core feature of schizophrenia and significant deficits are evident years prior to the manifestation of full psychotic symptoms [1, 2], escalating in severity during adolescence [3, 4]. Even though numerous studies have consistently reported a robust association between symptoms and cognitive impairment [5, 6], the association between these deficits and antipsychotics treatment response remains unclear. Antipsychotic drugs are the main treatment method for schizophrenia patients, it remains a significant challenge to assess a patient’s response to antipsychotics in clinical practice. The severity of cognitive deficits is aligned with levels of treatment resistance [7], therefore, an in-depth understanding the association between these deficits and treatment course can provide valuable insights into the clinical treatment and further prediction of the prognosis of schizophrenia.

A clear understanding of the longitudinal relationship between cognition dysfunction and symptoms is well established, schizophrenia research suggested that cognitive deficits related to negative symptoms and impairs everyday functioning [8, 9]. While it is unclear whether cognitive performance at baseline can predict clinical response to antipsychotic medication. Approximately 40% of patients do not achieve a satisfactory response to initial antipsychotic administration [10], and there is a notable absence of reliable biomarkers to predict outcomes. Due to the relative difficulty in conducting large-scale, controlled treatment trials, only a few prospective studies have examined the predictive effect of cognition on treatment response in in FES patients undergoing initial treatment with antipsychotic medications. A recent treatment‑resistant schizophrenia research revealed that antipsychotic resistance is associated with poorer cognitive function [7]. Another previous discriminant function analysis study discovered that verbal fluency can accurately discriminate 88% of the cases as responder or the non-responder group during a pharmacological treatment [11]. All these results suggest the severity of cognitive deficits maybe associated with treatment response and long-term prognosis.

The unresolved issue is whether predictive effect of cognition differs among different antipsychotics administration. A longitudinal study that included aripiprazole and risperidone to examined relationship of cognition to clinical response suggest that planning and reasoning skills in FES might hold prognostic value [12], however, the limited sample size restricts the comparison between different medications. Besides another unresolved question is which cognitive dimension can predict clinical response in FES. Patients with schizophrenia manifest deficits in attention, memory, executive function, and social cognition [13, 14]. Previous discriminant analysis revealed that verbal fluency can discriminate the responder from the non-responder group during a pharmacological treatment [11]. While another research found that baseline performance in the MCCB reasoning domain (reasoning/ problem solving) was a significant and robust predictor of response to antipsychotic treatment of week 12 [12]. The response of patients to antipsychotic medications is influenced by the baseline severity of schizophrenia, meaning that those with more severe initial symptoms often experience more pronounced symptom reduction [15]. Certain research suggests that the severity of initial symptoms may serve as a potential indicator for subsequent adjustments in antipsychotic treatment for individuals with schizophrenia [16]. While previous studies, limited by sample size, did not perform stratified analysis based on the severity of the disease.

Taken together, previous studies have not investigated the interactive effects of baseline cognition with antipsychotics and disease severity on clinical outcomes. Consequently, this study aims to determine whether the association between baseline cognition and clinical response is dependent on antipsychotics and severity of illness, with the ultimate goal of promoting individualized treatment and precision psychiatry.

## Methods

### Participants

This study was sourced from the inaugural subproject of a multicenter clinical trial aimed at optimizing and individualizing pharmacological treatment for individuals undergoing their initial episode of schizophrenia. All procedures were approved by the institutional review boards of the Second Xiangya Hospital of Central South University (ref: 2016S035). Patients were either antipsychotic-naïve or had discontinued short-term antipsychotic treatment for at least three months before enrollment. Schizophrenic patients were recruited from 10 sites and randomly assigned to six antipsychotic groups (olanzapine, risperidone, aripiprazole, ziprasidone, amisulpride, haloperidol). Owing to a reduction in available funding compared to the projected budget, the ziprasidone and haloperidol groups listed in the protocol registration were omitted from the final study. Antipsychotic medications were started at a low dosage and incrementally increased to reach the therapeutic dose within one week, as outlined in the study protocol, and maintained at that level following the titration phase. Daily doses of antipsychotics were recorded and converted to chlorpromazine equivalents [17]. Current study received approval from the Institutional Review Boards of the Second Xiangya Hospital of Central South University and the respective sites involved. Written informed consent was obtained from all participants.

**Fig. 1:**
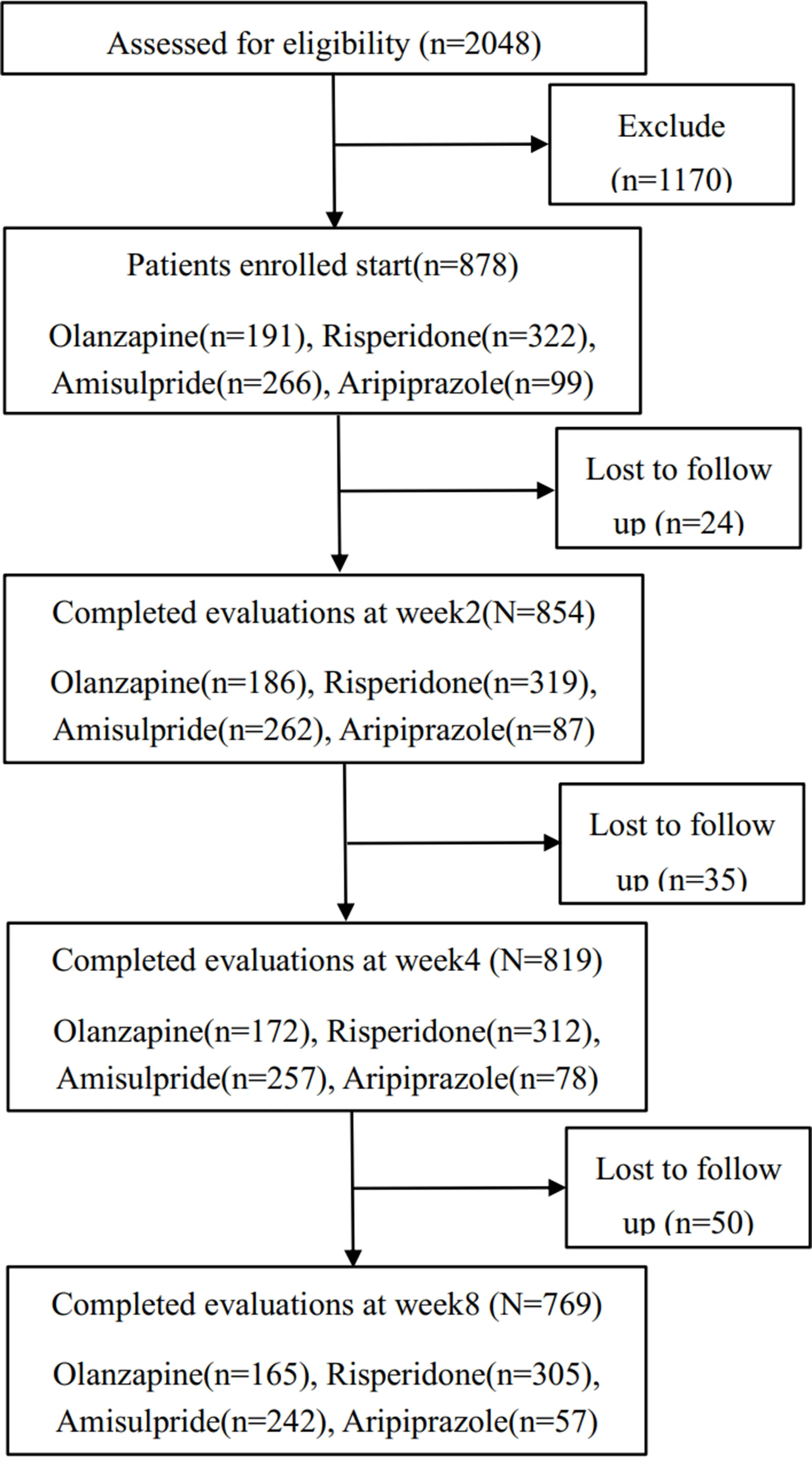
Flowchart of the study design

In these cohorts, the inclusion criteria included individuals aged between 18 and 65, experiencing their first or early psychotic episode of schizophrenia, and diagnosed with schizophrenia according to the criteria specified in the Diagnostic and Statistical Manual of Mental Disorders, Fifth Edition (DSM-5) or the International Classification of Diseases, Tenth Edition (ICD-10); participants must be experiencing a current episode of psychotic symptoms with a disease course < 3 years and intermission < 6 months. Exclusion criteria included individuals who: (1) with serious physical illness; (2) were pregnant or lactating; (3) had a current or previous DSM-5/ICD- 10 diagnosis of a substance use disorder, intellectual disability, or other specific systemic diseases; (4) suffered from a known or suspected clinically unstable systemic medical condition. Detailed information regarding this trial protocol has been previously published [18]. Initially, 2,048 patients were screened for eligibility. Subsequently, 1170 individuals were disqualified for either failing to meet the inclusion criteria or declining enrollment. The remaining 878 subjects were randomized to treatment with olanzapine, risperidone, aripiprazole, or amisulpride (See Fig. 1). Follow-up assessments were completed by 854 participants at week 2, 819 participants at week 4 and 769 at week 8.

### Measures

#### Clinical symptoms

Participants were evaluated and followed at four time points after titration (baseline, week 2, week 4, and week 8). Clinical symptom evaluations were conducted using the PANSS. All the assessors involved in this study received consistency training before the research began. Participants were stratified according to the severity of their illness at baseline. We followed the interpretation guidelines for the PANSS total scores and linked these scores to clinical global impression ratings using an anchor-based approach: mild, moderate, and severe schizophrenia correspond to PANSS total scores of 58–75, 76–95, and 95 or higher, respectively [19]. In the follow-up cohorts, the treatment response was evaluated at week 8. For participants in the follow-up cohort, The PANSS total score reduction rate was calculated as (baseline PANSS total score−follow-up PANSS total score)/ (baseline PANSS totalscore − 30) ×100% [20]. Patients were then classified into either a “response” or “non-response” group, depending on whether their PANSS reduction rate surpassed the 50% threshold at week 8, in accordance with predefined criteria [21].

#### Neurocognitive function assessments

Cognitive function was assessed using the MATRICS (Measurement and Treatment Research to Improve Cognition in Schizophrenia) Consensus Cognitive Battery (MCCB) [22, 23]. MCCB, which consists of 10 standardized cognitive measure dimensions to generate scores and a global composite score across seven cognitive domains, including processing speed (evaluated through symbol coding, category fluency, and the trail making test), attention/vigilance (assessed via the continuous performance test), working memory (measured using the digital sequence and Wechsler memory scale spatial span), verbal learning (evaluated with the Hopkins verbal learning test), visual learning (assessed through the brief visuospatial memory test), reasoning/problem solving (measured with the neuropsychological assessment battery), and social cognition (evaluated using the Mayer–Salovey–Caruso Emotional Intelligence Test). In alignment with previous studies, all cognitive scores were converted into T-scores following adjustments for age, gender, and level of education [22]. All raters received standardized training prior to the study and were blind to the treatment allocation.

### Statistical analysis

#### Comparison of demographics and clinical characteristics

Statistical analyses were performed in IBM SPSS Statistics version 23 (SPSS, Chicago, USA). Continuous variables were descried as means and standard deviations. Categorical variables were presented using frequencies and percentages. Data with normal distribution were compared by t-test or one-way analysis of variance (ANOVA) followed by Bonferroni’s post hoc multiple comparison test, while data with a non-normal distribution were analyzed using a 2-tailed Mann-Whitney U test to compare between two groups, or the Kruskal-Wallis test to compare multiple groups. The chi-square or Fisher’s exact test was used to test for differences in the distribution of categorical variables. The association between PANSS reduction rate and neurocognitive performance was explored via the pearson correlation analysis. All multiple comparisons were adjusted using the Bonferroni correction. Missing data were imputed using multiple imputation. Two-tailed p-values of < 0.05 were considered to be of statistical significance.

#### Logistic regression analysis

In order to preliminarily explore whether different levels of cognition are influencing factors in the response to antipsychotic medication, different dimensions of neurocognitive function, antipsychotic medication, baseline PANSS score, the interaction term between neurocognitive and antipsychotics, and the interaction term between baseline PANSS score and neurocognitive were considered as independent variables, with treatment response to 8-week antipsychotic medication as the dependent variable in logistic regression model. Meanwhile, age, gender, education level, illness duration and chlorpromazine equivalents were included in the logistic regression model as covariates. Variance Inflation Factors (VIF) was used to assess the collinearity between baseline disease severity and neurocognition. When interactions term in the logistic regression equation were significant, simple effects analysis was performed to explore the association between neurocognitive and treatment response across different disease subgroups. For the antipsychotic subgroups, covariates included age, gender, education level, chlorpromazine equivalents, illness duration, and baseline PANSS score. For the disease severity subgroups, the logistic regression model adjusted for age, gender, education level, chlorpromazine equivalents, illness duration, and different antipsychotic medications. All multiple comparisons were adjusted using the Bonferroni correction.

## Results

### Demographics and clinical characteristics

Demographic characteristics are displayed in Table 1. In total, 769 participants (men, 351 [50.6%]; women, 418 [49.4%]) with a median age of 26.47 ± 8.35 years and duration of illness were 2.26 ± 1.43 years were included. The mean baseline PANSS total score was 84.90 ± 16.08. Upon enrollment, the patient cohort comprised 176 individuals classified as severe, 424 as moderate, and 169 as mild in terms of disease severity. There were significant differences among the four groups in gender, education level, illness duration, chlorpromazine equivalents, and baseline PANSS scores (All *P* < 0.05), with the exception of age, which showed no significant difference.

**Table 1:** Demographic characteristics of the study population

Variables	Olanzapine (*N* = 165)	Risperidone (*N* = 305)	Amisulpride (*N* = 242)	Aripiprazole (*N* = 57)	*P*
Age (years)	26.84 ± 9.28	25.56 ± 8.12	27.02 ± 7.61	27.80 ± 9.22	0.091
Sex (male/female)	(77/88)	(134/171)	(125/117)	(15/42)	0.006
Education (year)	12.01 ± 2.96	11.54 ± 2.82	11.57 ± 3.82	13.96 ± 2.55	< 0.001
Illness duration(year)	2.25 ± 1.95	1.81 ± 1.39	2.84 ± 2.00	2.12 ± 1.94	< 0.001
Chlorpromazine equivalent dosage(mg/day)	217.34 ± 126.05	257.87 ± 120.12	272.62 ± 93.58	163.67 ± 71.48	0.001
PANSS total score	84.43 ± 19.58	86.98 ± 16.09	83.47 ± 12.20	81.09 ± 18.17	0.015
Severity of illness					
Severe schizophrenia (PANSS total score > 95)	36	79	51	10	
Moderate schizophrenia (95 ≥ PANSS total score > 75)	73	168	158	25	< 0.001
Mild schizophrenia (75 ≥ PANSS total score > 58)	56	58	33	22	

Data are presented as means ± standard deviations

Abbreviations: PANSS, Positive and Negative Syndrome Scale

### Correlations

**Fig. 2:**
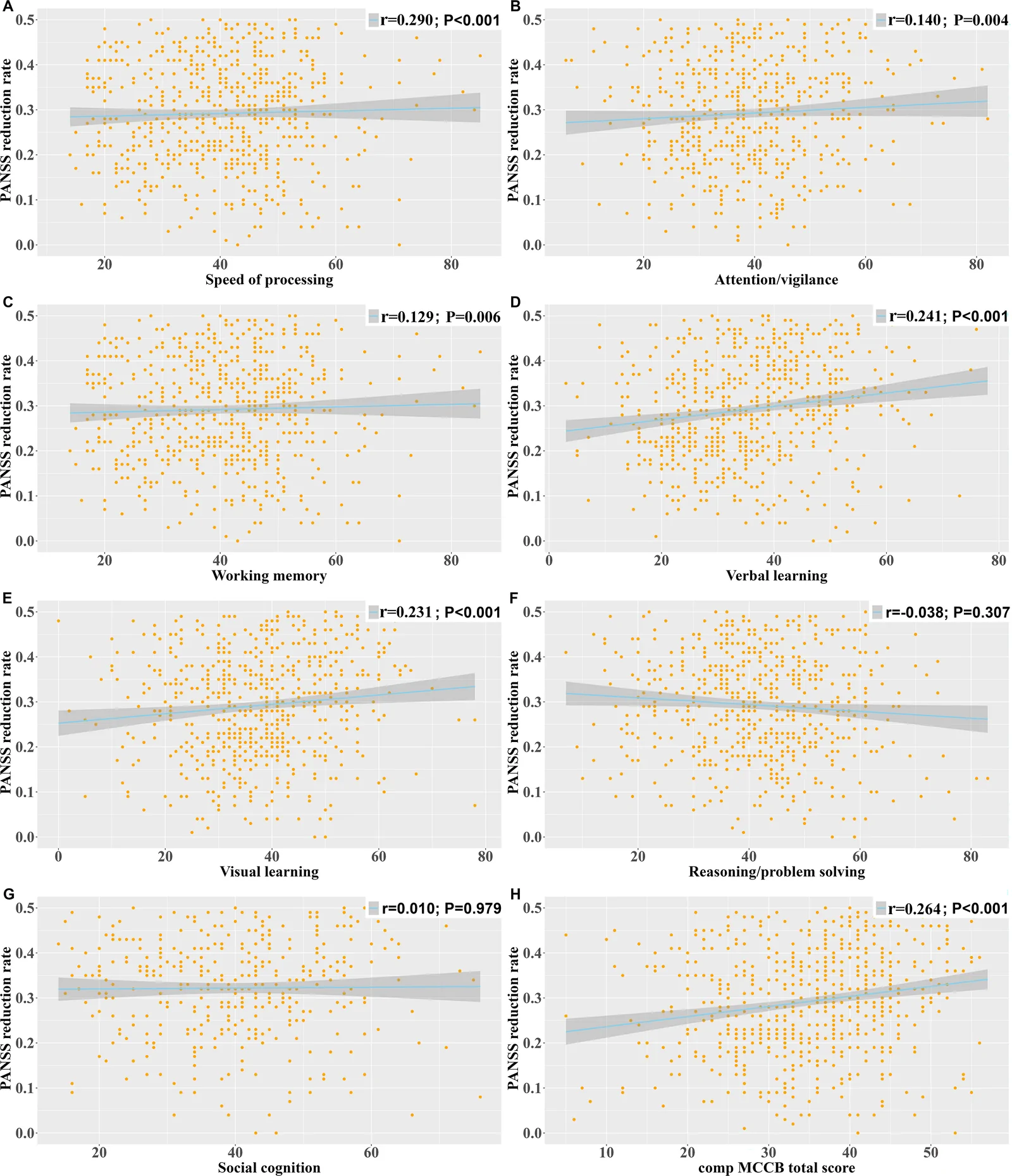
Correlations between baseline cognitive function and PANSS reduction rate at week 8. Abbreviations: PANSS, Positive and Negative Syndrome Scale; MCCB: the MATRICS Consensus Cognitive Battery. Corrected *P* values by controlling the familywise error at the 0.00625 (*P* < 0.05/8 was significant) level, using a Bonferroni correction

To explore the relationship between baseline cognition and treatment response, we initially conducted a correlation analysis between cognitive measures and the PANSS reduction rate. The 8-week PANSS reduction rate were associated with speed of processing (*r* = 0.290; *P* < 0.001), attention/vigilance (*r* = 0.140; *P* = 0.004), working memory (*r* = 0.129; *P* = 0.006), verbal learning (*r* = 0.241; *P* < 0.001), visual learning (*r* = 0.231; *P* < 0 0.001) and comp MCCB total score (*r* = 0.264; *P* < 0.001) (Fig. 2). All P values remained significant after Bonferroni correction.

### Baseline predictors of response

**Fig. 3:**
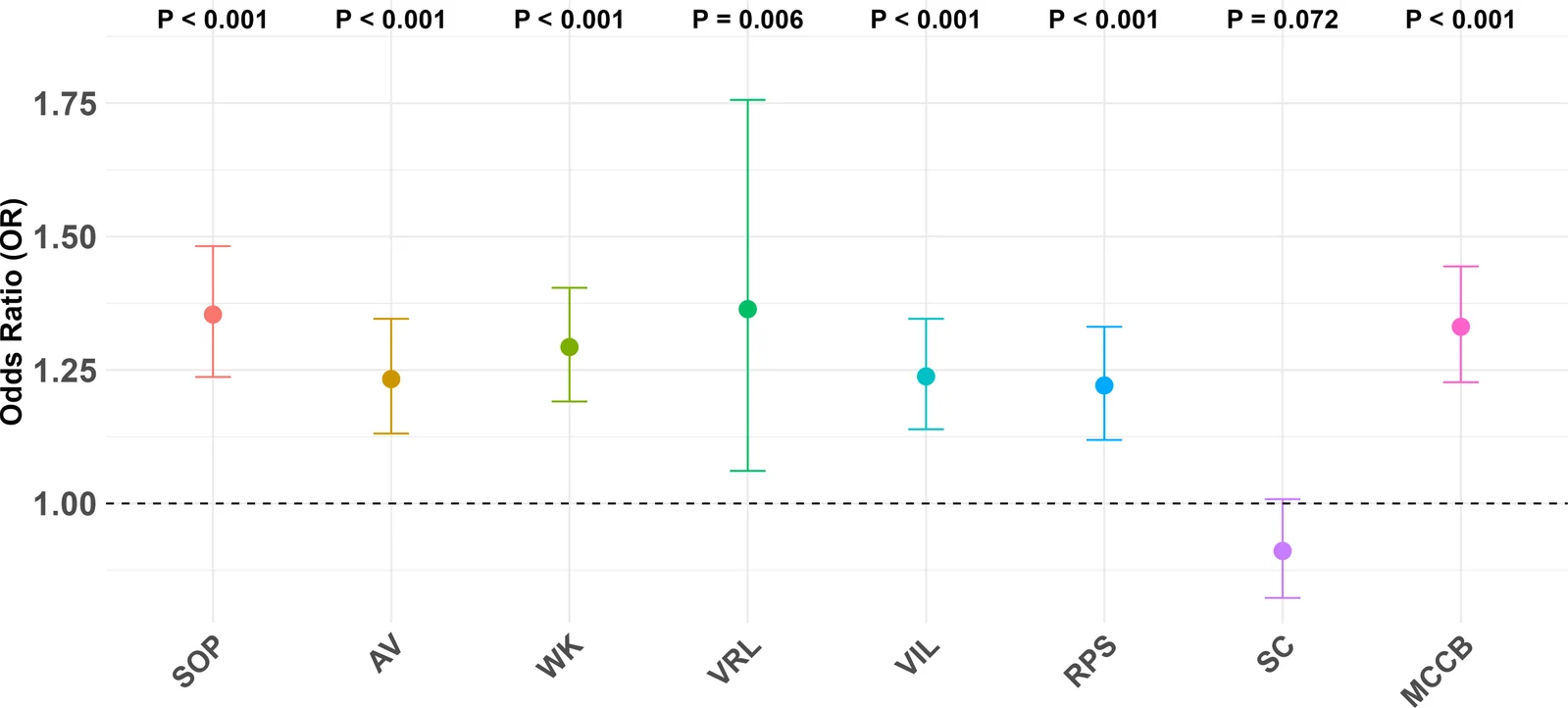
Adjusted odds ratios (aORs) and 95% confidence intervals for the association between baseline cognitive domains and clinical response. *P* values were two-sided and adjusted for age, gender, education level, illness duration, chlorpromazine equivalents, baseline PANSS total score and antipsychotic type; Corrected P values by controlling the familywise error at the 0.00625 (*P* < 0.05/8 was significant) level, using a Bonferroni correction. Abbreviations: SOP: Speed of Processing; AV: Auditory Verbal Learning; WK: Working Memory; VRL: Visual Reasoning and Learning; VIL: Visual Learning; RPS: Reasoning and Problem Solving; SC: Social Cognition; MCCB: MATRICS Consensus Cognitive Battery total score

We performed preliminary logistic regression analyses to investigate the association between different neurocognitive dimensions and treatment response to antipsychotics, regarding the neurocognitive as independent variables and the treatment responses as dependent variables, after controlling for age, gender, education level, illness duration, baseline PANSS scores, antipsychotics and chlorpromazine equivalent. Logistic regression analyses revealed that multiple cognitive dimensions were associated with treatment response to antipsychotic medications. Higher performance in comp MCCB total score was associated with superior clinical response week8(*aOR* = 1.331; *CI* = 1.227–1.444, *P* < 0.001) (Fig. 3). Further analysis of neurocognitive subdimensions revealed that superior performance in distinct neurocognitive dimensions, including speed of processing, attention/vigilance, working memory, verbal learning, visual learning reasoning and problem solving, except for social cognition were all significantly related to superior clinical response (all *P* < 0.05). We use the VIF to evaluate the degree of collinearity between disease severity and neurocognition. VIF values between all neurocognitive subdomains and baseline PANSS scores were less than 5(Supplementary Table 1). All P values remained significant after Bonferroni correction.

To examine whether the relationship between cognitive performance and clinical response was moderated by antipsychotics and severity of the disease, interaction terms were included in the logistic regression models. As shown in Table 2, significant interactions were observed for speed of processing×antipsychotics(*Wald χ2* = 20.535, *P* < 0.001) and speed of processing×PANSS baseline(*Wald χ2* = 16.321, *P* < 0.001); attention/Vigilance × antipsychotics (*Wald χ2* = 15.087, *P* = 0.002) and attention/Vigilance × PANSS baseline (*Waldχ2* = 31.194, *P* < 0.001); working memory×PANSS baseline(*Wald χ2* = 60.331, *P* < 0.001); verbal learning × antipsychotics (*Wald χ2* = 88.095, *P* < 0.001); visual learning × antipsychotics (*Wald χ2* = 54.801, *P* < 0.001); reasoning/problem solving × PANSS baseline (*Waldχ2* = 40.012, *P* < 0.001); comp MCCB total score × antipsychotics (*Wald χ2* = 30.876, *P* < 0.001) and comp MCCB total score × PANSS baseline (*Waldχ2* = 16.812, *P* < 0.001). No significant interaction effects were found for other cognitive domains (all *P* > 0.05). All P values remained significant after Bonferroni correction.

**Table 2:** Results of interaction models for clinical response: Effects of baseline cognition, PANSS baseline, and antipsychotics

MCCB	Antipsychotics			PANSS baseline	
	Wald χ2	*P*		Wald χ2	*P*
Speed of processing	20.535	< 0.001		16.321	< 0.001
Attention/vigilance	15.087	0.002		31.194	< 0.001
Working memory	5.926	0.114		60.331	< 0.001
Verbal learning	88.095	< 0.001		9.968	0.010
Visual learning	54.801	< 0.001		5.523	0.019
Reasoning/problem solving	1.562	0.688		40.012	< 0.001
comp MCCB total score	30.876	< 0.001		16.812	< 0.001

Corrected P values by controlling the familywise error at the 0.00714(*P* < 0.05/87was significant) level, using a Bonferroni correction

Abbreviations: PANSS, Positive and Negative Syndrome Scale; MCCB: the MATRICS Consensus Cognitive Battery

### Simple slope analysis

**Fig. 4:**
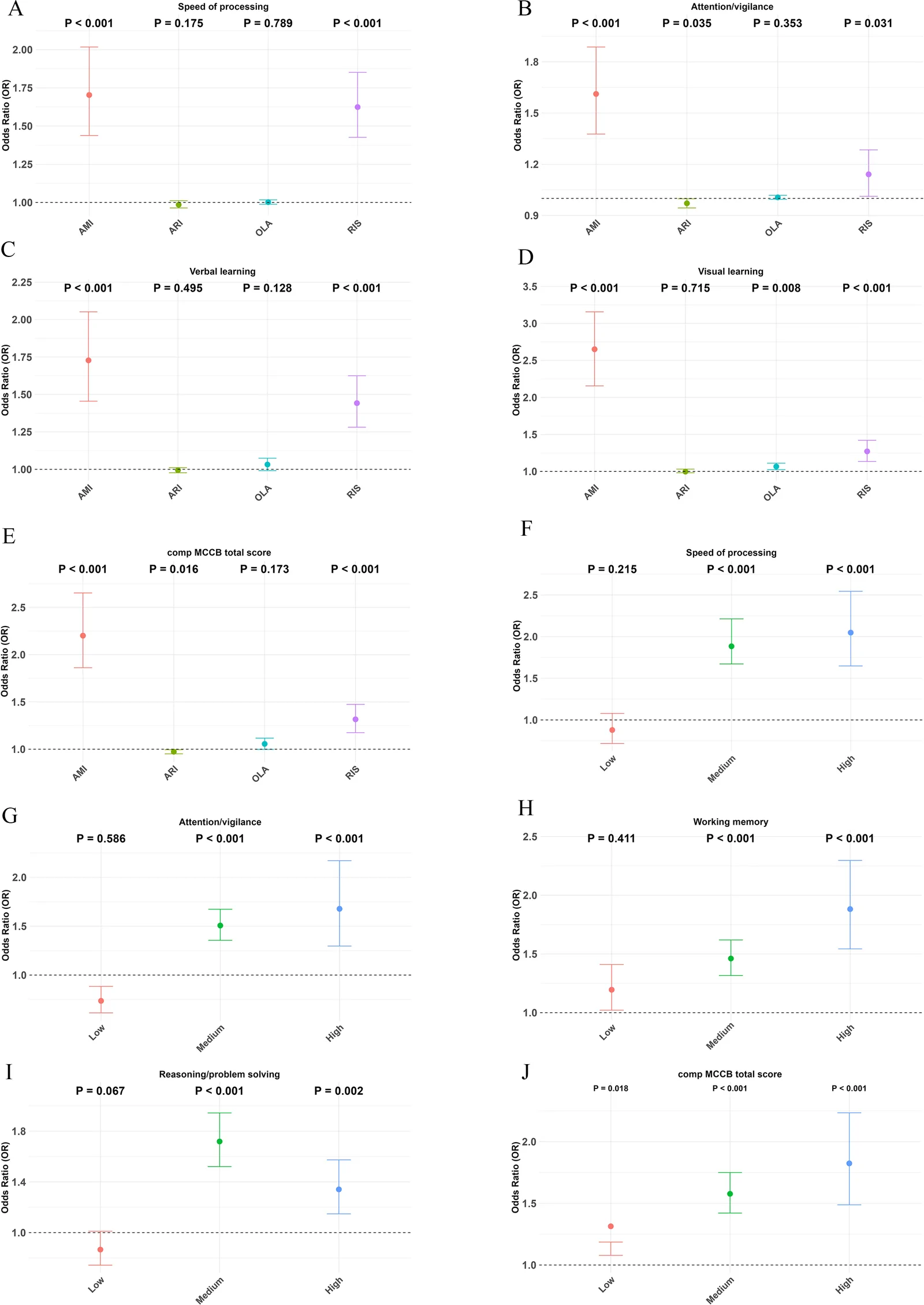
Stratified analysis of the association between baseline cognitive domains and treatment response, by antipsychotic medication type (**A–E**) and baseline illness severity (**F**–**J**). Odds ratios (ORs) and 95% confidence intervals are derived from logistic regression models adjusted for age, gender, education level, illness duration, chlorpromazine equivalents, baseline PANSS total score for antipsychotics stratified analysis; while adjusted for age, gender, education level, illness duration, chlorpromazine equivalents and antipsychotic medication type for baseline illness severity stratified analysis. Corrected P values by controlling the familywise error at the 0.00625 (*P* < 0.05/8 was significant) level, using a Bonferroni correction. Abbreviations: AMI: amisulpride; ARI: aripiprazole; OLA: olanzapine; RIS: risperidone

When stratified by baseline illness severity, our findings reveal that speed of processing(*aOR* = 1.884; *CI* = 1.672–2.213; *aOR* = 2.048; *CI* = 1.648–2.544 respectively), attention/vigilance(*aOR* = 1.507; *CI* = 1.356–1.674; *aOR* = 1.678; *CI* = 1.297–2.171 respectively), working memory(*aOR* = 1.460; *CI* = 1.316–1.619; *aOR* = 1.882; *CI* = 1.543–2.296 respectively), reasoning and problem solving(aOR = *1.719*; *CI* = 1.520–1.944; *aOR* = 1.340; *CI* = 1.148–1.574 respectively), and comp MCCB total score(*aOR* = 1.577; *CI* = 1.421–1.750; aOR = 1.824; *CI* = 1.488–2.234 respectively) were significantly positively correlated with clinical response in patients with medium -to-severe severity(Fig. 4).

Stratified analysis by antipsychotics revealed that speed of processing (*aOR* = 1.624; *CI* = 1.426–1.851; *aOR* = 1.703; *CI* = 1.438–2.017 respectively), verbal learning(*aOR* = 1.442; *CI* = 1.280–1.625; *aOR* = 1.835; *CI* = 1.556–2.163 respectively), visual Learning(*aOR* = 1.270; *CI* = 1.134–1.421; *aOR* = 2.650; *CI* = 2.156–3.157 respectively) and the comp MCCB total score(*aOR* = 1.316; *CI* = 1.175–1.474; *aOR* = 2.201; *CI* = 1.862–2.653 respectively) were primarily associated with the treatment response to risperidone and amisulpride, while attention/vigilance was significantly associated with treatment response to the amisulpride alone(*aOR* = 1.612; *CI* = 1.377–1.887). All these *P* values were adjusted using the Bonferroni correction.

## Discussion

In this study, we examined associations between baseline cognition and antipsychotic treatment response in a large sample of FES patients. We found that better cognitive performances at baseline, which was driven by performance on different cognitive domains, was associated with a superior clinical response to antipsychotics. Additionally, this association exhibited significant heterogeneity among patients with different antipsychotic administration and baseline disease severity.

The most striking result of our study was the ability of baseline superior cognitive performance in different cognitive subtests of MCCB was significantly associated with 8-week clinical response to antipsychotics, although the strength of the associations is modest, we interpret these findings with caution. Simple bivariate correlations often underestimate the true clinical utility of a variable by failing to account for the contribution of other clinical factors. Indeed, after adjusting for key covariates in our regression models, cognition emerged as a significant determinant of clinical response. This suggests that while the bivariate effect size is modest, baseline cognition may provide valuable clinical prognostic information. Current result was consistent with previously researches which reported associations between clinical treatment response and baseline cognitive performance, including attention [24], verbal memory [25], planning and reasoning [12, 26]. It is worth noting that in previous studies, differing sample characteristics and test batteries, as well as smaller sample sizes may account for the failure to converge on a single prognostic measure, the current study, with its larger sample size, addresses this limitation, indicating that most neurocognitive dimensions, except for social cognition, are significantly associated with superior clinical response. Collectively, these studies strongly indicate that baseline brief paper-and-pencil measures of neurocognitive functioning may provide significant prognostic information.

A critical methodological issue when interpreting these results is the potential for collinearity between baseline cognitive performance and symptom severity, given their simultaneous inclusion as covariates. Our diagnostics, however, showed VIF values for all neurocognitive subdomains and baseline PANSS scores to be less than 5, placing multicollinearity well within an acceptable range. This evidence supports the robustness of our findings, confirming that the independent contribution of cognition to clinical response is unlikely to be a statistical artifact arising from correlations with baseline symptoms.

Current research suggests that the association between most neurocognitive dimensions and clinical response may vary depending on the type of antipsychotics. Specifically, neurocognition is significantly associated with the clinical response to risperidone and amisulpride, but not with olanzapine and aripiprazole. Notably, the distribution of illness duration was not balanced across the different treatment groups. Given that previous studies have established a correlation between illness duration and cognitive [27], this baseline heterogeneity theoretically posed a risk of confounding bias in the analysis of the association between cognition and clinical response. To mitigate this confounding effect, the duration of illness was included as a covariate in all regression models. Despite the known influence of illness duration on cognition, the adjusted analysis continued to reveal heterogeneity in the predictive effect of baseline cognition on clinical response across different antipsychotic groups, suggesting that the observed findings are not attributable to differences in illness duration. A recent study of FES patients revealed that baseline MCCB reasoning subtest can effectively predict12-week clinical response to risperidone or aripiprazole, however, this study did not conduct further subgroup analyses based on antipsychotic drug subgroups (aripiprazole and risperidone) due to the small sample size [12]. Moreover, some previous small-scale studies did not restrict the types of antipsychotic medications, making it difficult to accurately determine whether the predictive effect of baseline cognition was influenced by the specific drug types [24, 25]. Olanzapine and risperidone have been reported to produce significant improvements in neurocognition in early psychosis patients, especially in schizophrenia patients with prominent negative symptoms [28]. These cognitive benefits were considered to relate to the improvement of clinical symptoms, theoretically, should effectively predict clinical response. However, the current study only detected that baseline cognition effectively predicts the clinical response to risperidone, but not to olanzapine.

The predominant hypothesis for cognitive impairment in schizophrenia involves hypofunction of the dopaminergic system in the prefrontal cortex [29, 30]. The observed heterogeneity in the association between baseline cognition and clinical response among various medications may be intimately tied to the specific pharmacological properties of different antipsychotics [31]. Amisulpride exhibits robust correlations between multiple cognitive dimensions and clinical response, which can be attributed to its unique mechanism of presynaptic D2/D3 autoreceptor regulation [32]. By blocking autoreceptor inhibition, amisulpride enhances endogenous dopamine release; this implies that its therapeutic efficacy is critically contingent upon the patient’s residual dopaminergic reserve. Specifically, this reserve is mirrored by baseline cognitive performance. Consequently, superior baseline cognition implies greater integrity of the prefrontal dopaminergic system, thereby amplifying the therapeutic efficacy of amisulpride and resulting in a strong correlation. Current findings indicate that the association between multiple cognitive dimensions and clinical response to risperidone is second only to that observed with amisulpride, whereas the association between clinical response to olanzapine and baseline cognition is relatively weak. Both olanzapine and risperidone enhance prefrontal dopamine release by blocking 5-hydroxytryptamine 2 A (5-HT₂A) receptors [33], thereby disinhibiting dopaminergic neurons. While this mechanism is effective, it lacks the precision of amisulpride’s direct presynaptic regulation. Specifically, although olanzapine also increases prefrontal dopamine, it possesses potent direct antihistaminic and anticholinergic properties [34]. These strong antihistaminic (H1) and anticholinergic (M1) effects generate non-specific sedation and stabilization. Consequently, even patients lacking dopaminergic reserve (poor baseline cognition) can achieve symptomatic improvement through these alternative pharmacological pathways. This efficacy, driven by non-cognitive mechanisms, dilutes the specific association between baseline cognition and clinical response. Another possible explanation is that in the current study, the sample sizes for olanzapine and aripiprazole were smaller compared to those for risperidone and amisulpride, which may have affected the accuracy of the results.

In addition, the current study conducted further subgroup analyses based on baseline disease severity, and the results suggest that the association between baseline cognition and treatment response varied with respect to baseline disease severity. Furthermore, the current study performed subgroup analyses stratified by baseline disease severity, revealing that the association between baseline cognition and treatment response is moderated by symptom severity. Specifically, correlations between treatment response and most neurocognitive domains—namely processing speed, attention/vigilance, working memory, and reasoning and problem solving—were significant primarily among patients with medium -to-severe symptoms. In contrast, these correlations were attenuated in patients with milder symptoms. Notably, clinical response remained robustly associated with visual and verbal learning irrespective of baseline severity. These observed discrepancies may be attributable to the neurobiological specificity inherent to distinct cognitive dimensions. Speed of processing, attention/vigilance, working memory, and reasoning/problem solving rely predominantly on prefrontal cortex dopaminergic function—mechanisms highly sensitive to dopamine levels and mediated via circuits involving the dorsolateral prefrontal cortex and the prefronto-striato-thalamic pathway [35–37]. In patients with mild symptom severity, the dopaminergic system is relatively preserved, and impairment has not yet breached the threshold necessary to disrupt these functions. Consequently, pharmacological interventions targeting this system yield limited incremental benefits in this cohort. Conversely, patients with medium -to-severe presentation exhibit a pronounced prefrontal dopamine ‘deficit.’ In this context, the restorative effects of pharmacological augmentation exhibit a linear relationship with the magnitude of the baseline deficit (i.e., severity of impairment). Thus, lower baseline scores indicate a greater potential for functional restitution, manifesting as a robust correlation with clinical response. In contrast, visual and verbal learning and memory are subserved primarily by the hippocampus and medial temporal lobe system [38, 39]. Manifestations of hippocampal volume reduction and functional aberration in these regions represent relatively stable neurobiological traits that persist across the schizophrenia disease course, regardless of stage or severity. Therefore, visual and verbal learning abilities may function as trait-like markers, where baseline levels reflect a stable structural state rather than a fluctuating symptom-dependent condition.

The current study had some notable limitations. This study included only four antipsychotics, further comparisons between drugs were unavailable. And the sample size of the aripiprazole group is small, future studies should expand the sample size and include a wider variety of antipsychotic drugs. Additionally, the follow-up period in the current study is limited to 8 weeks, the 8-week follow-up period limits our ability to draw definitive conclusions regarding sustained response or treatment resistance. Although previous studies have investigated longer-term outcomes, their sample sizes were relatively small. It is important to acknowledge that prior short-term antipsychotic use and a prolonged duration of illness in certain participants represent potential confounders. These variables may impact cognitive performance and subsequently affect the generalizability of our results. Considering that cognitive impairments observed in patients may be influenced by their intelligence Quotient (IQ), another limitation is the lack of IQ measurements in our study. Including IQ assessment as a confounding factor, may affect the research results. Additionally, due to the lack of individual item-level data for the PANSS, we could not perform separate analyses for these specific cognitive-related items, future studies should include these granular assessments to further clarify these associations.

## Conclusions

This a large multicenter, open-label, randomized trial provides findings suggesting that baseline cognitive performance is significantly associated with the clinical response to antipsychotic medications. The strength of these associations is influenced by the type of antipsychotics administration and the baseline severity. The present results suggest that cognitive performance in baseline might hold prognostic value in helping to determine which patients are likely to rapidly benefit from antipsychotic treatment.

## Supplementary Information

Below is the link to the electronic supplementary material.

Supplementary Material 1

## Data Availability

The datasets generated and/or analysed during the current study are not publicly available due to limitations of ethical approval involving the patient data and anonymity but are available from the corresponding author on reasonable request.

## Abbreviations

FES: First-episode schizophrenia
PANSS: The positive and negative syndrome scale
MCCB: MATRICS Consensus Cognitive Battery
DSM-5: Diagnostic and Statistical Manual of Mental Disorders, Fifth Edition
ICD-10: The International Classification of Diseases, Tenth Edition
IQ: Intelligence Quotient
