# Supplementary material for: Association between baseline cognition and clinical response in first-episode schizophrenia
Source: BMC Psychiatry. 2026 Aug 5;26:855. doi: 10.1186/s12888-026-08481-4 (PMC13726290; doi:10.1186/s12888-026-08481-4)
Supplement: Supplementary file 1 [file 12888_2026_8481_MOESM1_ESM.docx]

| MCCB Domains | Model independent variable | VIF |
| --- | --- | --- |
| Speed of processing | Speed of processing | 1.210 |
|  | Baseline PANSS | 1.069 |
| Attention/ vigilance | Attention/ vigilance | 1.057 |
|  | Baseline PANSS | 1.104 |
| Working memory | Working memory | 1.103 |
|  | Baseline PANSS | 1.076 |
| Verbal learning | Verbal learning | 1.107 |
|  | Baseline PANSS | 1.070 |
| Visual learning | Visual learning | 1.012 |
|  | Baseline PANSS | 1.070 |
| Reasoning/problem solving | Reasoning/problem solving | 1.173 |
|  | Baseline PANSS | 1.074 |
| Social cognition | Social cognition | 1.098 |
|  | Baseline PANSS | 1.160 |
| comp MCCB total score | comp MCCB total score | 1.092 |
|  | Baseline PANSS | 1.070 |

Supplementary Table1. Multicollinearity diagnostics for baseline PANSS scores and neurocognitive dimensions

Abbreviations: PANSS, Positive and Negative Syndrome Scale; MCCB: the MATRICS Consensus Cognitive Battery；VIF：Variance Inflation Factor.
